# Supplementary material for: Roles of H2S and NO in regulating the antioxidant system of Vibrio alginolyticus under norfloxacin stress
Source: PeerJ. 2021 Oct 5;9:e12255. doi: 10.7717/peerj.12255 (PMC8500093; doi:10.7717/peerj.12255)
Supplement: Supplemental Information 2 [file peerj-09-12255-s002.docx]

**DNA sequence data for table 1 in study**

1 ATGTTAAAAGGATTGCTATGCATTTCAGTTGCATCCGCTCTTGTCGCCGCCCCTAACGCGCTAGCAAAAACGGTAGAAATGACTGCTCTC 90

M L K G L L C I S V A S A L V A A P N A L A K T V E M T A L

91 AACACCAATGAGAGTGCGGGTACCATTGAGATTTCACAAAGTGATTATGGCGTTGTTTTTACGCCTCAATTATCAGGTTTGTCAGCAGGT 180

N T N E S A G T I E I S Q S D Y G V V F T P Q L S G L S A G

181 GTGCACGGTTTCCATATTCACACTAATCCATCTTGCGATAGCATAGAAAAGGATGACAAAACCGTATTAGGCGGCGCAGCGGGTGGCCAT 270

V H G F H I H T N P S C D S I E K D D K T V L G G A A G G H

271 TATGATCCTAACAACACAGGGAAGCATGGCTATCCTTGGACCGATGACAATCATTTAGGCGACTTGCCTCCACTCTATGTGGACATGGAC 360

Y D P N N T G K H G Y P W T D D N H L G D L P P L Y V D M D

361 GGCAATGCCAATCAGCCCGTTGTCGCTCCTCGCCTAAAATTATCGGATTTAAAAGGTCGAGCGTTAATGATCCATGCAGGTGGTGACAAT 450

G N A N Q P V V A P R L K L S D L K G R A L M I H A G G D N

451 CACTCCGATCACCCAAGTAAGCTAGGTGGTGGCGGCGCTCGCGTCGTATGTGGTGTTATTGAATAA 609

H S D H P S K L G G G G A R V V C G V I E *

**Figure 2-1 Open reading frame and deduced amino acid sequences of *SOD* gene in *Vibrio alginolyticus* HY9901**

1 ATTCGCTTGGCTCCGCAAAAAGATTGGCAAGGGAACGAACCTGAACGTCTAAGCCGTGTTCTTGCGGTGTTAGAAAAAATAGCAGCAGAA 90

I R L A P Q K D W Q G N E P E R L S R V L A V L E K I A A E

91 GAGGGCTGTAGTGTTGCAGATGCTATCGTGCTTGCTGGTAACGTAGGTATTGAACTGGCTGCGCGTGCCGCAGGTCATGATGTATCTGTG 180

E G C S V A D A I V L A G N V G I E L A A R A A G H D V S V

181 CCATTCGCGCCAGGCCGAGGTGATGCCACCCAAGAGATGACGGATGTTGAATCATTCGAAGTACTTGAGCCTGTTGCTGACGGTTTCCGA 270

P F A P G R G D A T Q E M T D V E S F E V L E P V A D G F R

271 AATTGGTTGAAGAAAGACTACGTGGTTAAGCCAGAAGAGCTTTTATTAGATCGTGCTCAGCTTATGGGGCTAACGGCGCCAGAAATGACG 360

N W L K K D Y V V K P E E L L L D R A Q L M G L T A P E M T

361 GTACTTATCGGTGGTATGCGCGTTCTAGGCAGTAACTACGGCGGTGGAAAAGAGGGCGTATTTACTGACCGAGTCGGCACTTTGTCGAAT 450

V L I G G M R V L G S N Y G G G K E G V F T D R V G T L S N

451 GATTTCTTCGTGAACCTTACTGACATGTCATATGCGTGGCAACCAGTAAGTGCTAATCAGTACGAAATCCGTGATCGTAAAACCGACGCT 540

D F F V N L T D M S Y A W Q P V S A N Q Y E I R D R K T D A

541 GTGAAGTGGACGGCAACCCGTGTGGATCTTGTCTTTGGTTCAAACTCGATCCTGCGTTCGTATGCGGAAGTGTATGCGCAAGATGATAAT 630

V K W T A T R V D L V F G S N S I L R S Y A E V Y A Q D D N

631 CAGGAAAAATTCATCCATGACTTCATCGCCGCATGGACGAAAGTCATGAACGCGGATCGTTTTGACTTGCAATAA 705

Q E K F I H D F I A A W T K V M N A D R F D L Q *

**Figure 2-2 Open reading frame and deduced amino acid sequences of *CAT* gene in *Vibrio alginolyticus* HY9901**

1 ATGGCGACTCATTTTGACTATATCTGTATCGGTGGCGGCAGTGGCGGCATCGCATCTGCAAACCGTGCGGCGATGTACGGCGCGAAAGTT 90

M A T H F D Y I C I G G G S G G I A S A N R A A M Y G A K V

91 GCGCTAATCGAAGCTCAAGACCTTGGCGGTACCTGTGTAAACGTAGGTTGTGTACCGAAAAAAGTGATGTGGCATGGCGCGCAAATCGCA 180

A L I E A Q D L G G T C V N V G C V P K K V M W H G A Q I A

181 GAAGCAATGAACCTGTACGCAGAAGATTACGGTTTTGATGTCGACGTGAAAGGTTTCGACTGGAGCAAACTGGTTGAGAGCCGTCAGGCA 270

E A M N L Y A E D Y G F D V D V K G F D W S K L V E S R Q A

271 TACATCGGTCGTATTCACCAATCTTACGATCGCGTTCTTGGCAACAACAAAGTAAACGTGATTAAAGGCTTTGCTAAGTTTGTTGACGAA 360

Y I G R I H Q S Y D R V L G N N K V N V I K G F A K F V D E

361 AAAACTGTCGAAGTAAACGGTGAACACTACACAGCAGATCACATCCTGATCGCGGTTGGTGGTCGTCCTACTATTCCAAACATTCCAGGC 450

K T V E V N G E H Y T A D H I L I A V G G R P T I P N I P G

451 GCTGAATACGGCATCGATTCAAACGGCTTCTTCGACCTAGCAGAGCAACCAAAACGCGTTGCGGTTGTGGGTGCTGGCTACATCGCAGTT 540

A E Y G I D S N G F F D L A E Q P K R V A V V G A G Y I A V

541 GAAATTGCTGGCGTACTGCACGCGCTAGGTACAGAAACGCACCTGTTCGTACGTAAAGAGTCACCACTGCGTAGCTTCGATCCAATGATC 630

E I A G V L H A L G T E T H L F V R K E S P L R S F D P M I

631 ATCGATACGTTGGTTGAAGTGATGGACGCAGAAGGTCCAAAACTGCACACCCACTCTGTACCAAAAGAAGTGGTAAAAGAAGCCGATGGC 720

I D T L V E V M D A E G P K L H T H S V P K E V V K E A D G

721 AGCCTAACTCTGCACCTAGAAAACGGTGAAAGCCAAAACGTTGACCAACTGATTTGGGCAATCGGTCGTCACCCAGCAACTGACGCAATC 810

S L T L H L E N G E S Q N V D Q L I W A I G R H P A T D A I

811 AACCTAGCGTCAACTGGTGTTGCAACGAACGAAAAAGGCTACATTAAGGTAGACGAATACCAAGAAACCAACGTGAAAGGCATCTACTGT 900

N L A S T G V A T N E K G Y I K V D E Y Q E T N V K G I Y C

901 GTGGGTGACATCATGGAAGGCGGTATCGAGCTAACACCAGTAGCAGTGAAAGCGGGTCGTCAACTTTCTGAGCGTTTGTTCAACAACAAA 990

V G D I M E G G I E L T P V A V K A G R Q L S E R L F N N K

991 CCAAACGCGAAGATGGACTACGATTTGGTACCAACTGTGGTATTTAGCCACCCACCAATCGGCACTATCGGTCTTACAACGCAAGAAGCG 1080

P N A K M D Y D L V P T V V F S H P P I G T I G L T T Q E A

1081 GAAGAGAAGTACGGCAAAGACAACATCAAAGTCTACACATCTGGCTTCACGGCGATGTACACCGCTGTTACTAAGCACCGTCAACCATGT 1170

E E K Y G K D N I K V Y T S G F T A M Y T A V T K H R Q P C

1171 AAGATGAAGCTTGTATGTGCGGGTGAAGAAGAAACGGTTGTTGGTCTACACGGCATCGGTTTCACGGTAGATGAAATGATCCAAGGCTTC 1260

K M K L V C A G E E E T V V G L H G I G F T V D E M I Q G F

1261 GGCGTAGCAATGAAGATGGGCGCAACTAAAGCAGACTTCGACTCTGTTGTGGCTATCCACCCAACGGGCTCAGAAGAGTTCGTAACGATG 1350

G V A M K M G A T K A D F D S V V A I H P T G S E E F V T M

1351 CGTTAA 1356

R *

**Figure 2-3 Open reading frame and deduced amino acid sequences of *GR* gene in *Vibrio alginolyticus* HY9901**
